# Comparative effects of ultrasonic and conventional sealer placement techniques on endodontic sealer layer quality: a systematic review and meta-analysis of *in vitro* studies

**DOI:** 10.3389/fdmed.2026.1909391

**Published:** 2026-08-25

**Authors:** Avishikta Banerjee, Niranjan Harikrishna, Aritro Bhattacharyya, Nishmitha Hegde, Mithra N. Hegde

**Affiliations:** 1 Department of Conservative Dentistry and Endodontics, NITTE (Deemed to be University) AB Shetty Memorial Institute of Dental Sciences, Mangaluru, India; 2 Centre for Public Policy, Indian Institute of Management Bangalore, Bengaluru, India

**Keywords:** dental cements, endodontic sealer, root canal obturation, root canal therapy, ultrasonics

## Abstract

**Aim:**

This systematic review aims to critically appraise the existing literature on the influence of ultrasonic sealer placement techniques compared to other sealer placement methods on the quality of the sealer layer coated on root canal walls. The sealer layer quality was assessed based on the depth of sealer penetration into dentinal tubules, the extent of canal wall coverage with sealer, interfacial sealer adaptation, void formation, and push-out bond strength.

**Methodology:**

A comprehensive literature search was conducted in PubMed, Scopus, and Web of Science, following the PRISMA guidelines, covering studies published between 2000 and 2026. *In vitro* studies comparing ultrasonic and non-ultrasonic sealer placement techniques, assessing the sealer layer quality, were included. 7,007 records were initially identified, of which 10 studies met the eligibility criteria. A standardised form was used for data extraction, and the risk of bias was assessed using the Quality Assessment Tool for *in-vitro* Studies (QUIN). A two-level random-effects meta-analysis (REML) was performed using the metafor package in R for sealer penetration depth and percentage of canal wall coverage outcomes.

**Results:**

10 *in vitro* studies were assessed in the systematic review, of which 6 were included in the meta-analysis. Meta-analysis of sealer penetration depth (*k* = 17; 3 studies) yielded a pooled WMD of 254.66 µm (95% CI: 180.88–328.44; *p* < 0.001; *I*^2^ = 100%) favouring ultrasonic activation. For the percentage of canal wall coverage (*k* = 27; 4 studies), the pooled WMD was 17.79% (95% CI: 9.73–25.86; *p* < 0.001; *I*^2^ = 99.1%), also favouring ultrasonic activation. Qualitative synthesis further indicated more favourable interfacial adaptation, reduced void formation, and higher push-out bond strength with ultrasonic sealer placement.

**Conclusions:**

Within the limitations of laboratory evidence, ultrasonic methods of sealer placement were associated with improved sealer layer quality compared with other methods; however, given the substantial heterogeneity among included studies and the exclusively *in vitro* nature of the evidence, these findings should not be interpreted as indicating clinical superiority without further clinical validation.

**Systematic Review Registration:**

https://www.crd.york.ac.uk/PROSPERO/view/CRD420261303375, identifier PROSPERO: CRD420261303375.

## Introduction

The principal objective of obturation in endodontics is to achieve a three-dimensional, fluid-impervious seal that eliminates niches for bacterial growth and reduces the risks of an endodontic reinfection (1–3). Gutta-percha, which is routinely used as a core filling material in endodontic obturation, is non-adherent to dentin (4). Endodontic sealers serve as an intermediary layer between the gutta-percha and root canal dentin, filling the voids between them, and contributing to a better endodontic seal (5).

The quality of the sealer layer placed hence plays a pivotal role in determining obturation success. An ideal sealer layer should be thin, void-free, well-adapted to the dentinal walls, and exhibit penetration into the dentinal tubules (6). The quality of the sealer layer coated on the root canal walls is influenced by the design and mechanism of action of the sealer-placing instruments (7). Conventional sealer placement methods, such as manual application with K-files, gutta-percha points, or paper points, primarily rely on passive coating of the canal walls. Studies have shown that these techniques result in an inconsistent sealer layer with voids, limited penetration into dentinal tubules, and suboptimal adaptation to canal walls (6, 8). To address these limitations, activation-based sealer placement techniques were introduced. Lentulo spirals function in a rotatory fashion, coating dentinal walls with sealer due to the centrifugal forces generated (9). Sonic and ultrasonic activation aim to enhance sealer flow and distribution through acoustic streaming and hydrodynamic agitation, potentially improving sealer penetration and adaptation along the dentinal interface. Several laboratory investigations have evaluated these techniques; however, their findings vary with respect to the extent of improvement observed across different outcome parameters and sealer types (8, 10, 11).

Given the growing body of *in vitro* evidence and the lack of consensus regarding the comparative effectiveness of ultrasonic sealer placement, a systematic review and synthesis of available data was warranted. Therefore, this systematic review was designed using a PICO framework to evaluate laboratory studies assessing the sealer layer formed within instrumented root canals (Population), comparing ultrasonic sealer placement techniques (Intervention) with other sealer placement methods (Comparator), and analysing outcomes related to depth of sealer penetration, percentage of canal wall coverage, marginal adaptation, void formation, and push-out bond strength (Outcomes).

## Materials and methods

This systematic review was registered in PROSPERO: CRD420261303375.

### Eligibility criteria

*In vitro* or laboratory-based experimental studies that evaluated a sealer layer placed within instrumented human root canals were included. The studies were considered eligible for inclusion if ultrasonic devices for sealer placement were evaluated in comparison to at least one non-ultrasonic sealer placement technique, such as manual application using hand files, paper points, or gutta-percha points; rotary sealer placement using Lentulo spirals, or sonic activation of sealer. Eligible studies were required to assess the quality of the sealer layer using quantitative or qualitative outcome measures such as the depth of sealer penetration into dentinal tubules, extent of canal wall coverage with sealer, marginal adaptation, presence of voids, and push-out bond strength. Only studies conducted on extracted human teeth were included. Furthermore, eligibility was restricted to full-text articles published in peer-reviewed journals, written in English, and published between 2000 and 2026.

Clinical studies, case reports, case series, animal studies, and *in vitro* investigations using non-human extracted teeth or artificial canal models, including resin blocks, were excluded. Narrative reviews, systematic reviews, meta-analyses, editorials, letters to the editor, and conference abstracts were also excluded. Studies that did not evaluate ultrasonic sealer placement or activation, or that lacked a comparator group by assessing only a single sealer placement technique, were excluded. Studies were excluded if they did not assess outcomes related to sealer layer quality, including sealer penetration, canal wall coverage, marginal adaptation, void formation, or bond strength. Investigations focusing solely on the physical, chemical, or biological properties of sealers without evaluating the influence of sealer placement techniques were excluded. Studies with insufficient methodological detail regarding sealer placement or activation protocols and incomplete or non-extractable outcome data were also excluded.

### Literature search strategy and screening procedure

**Figure 1:**
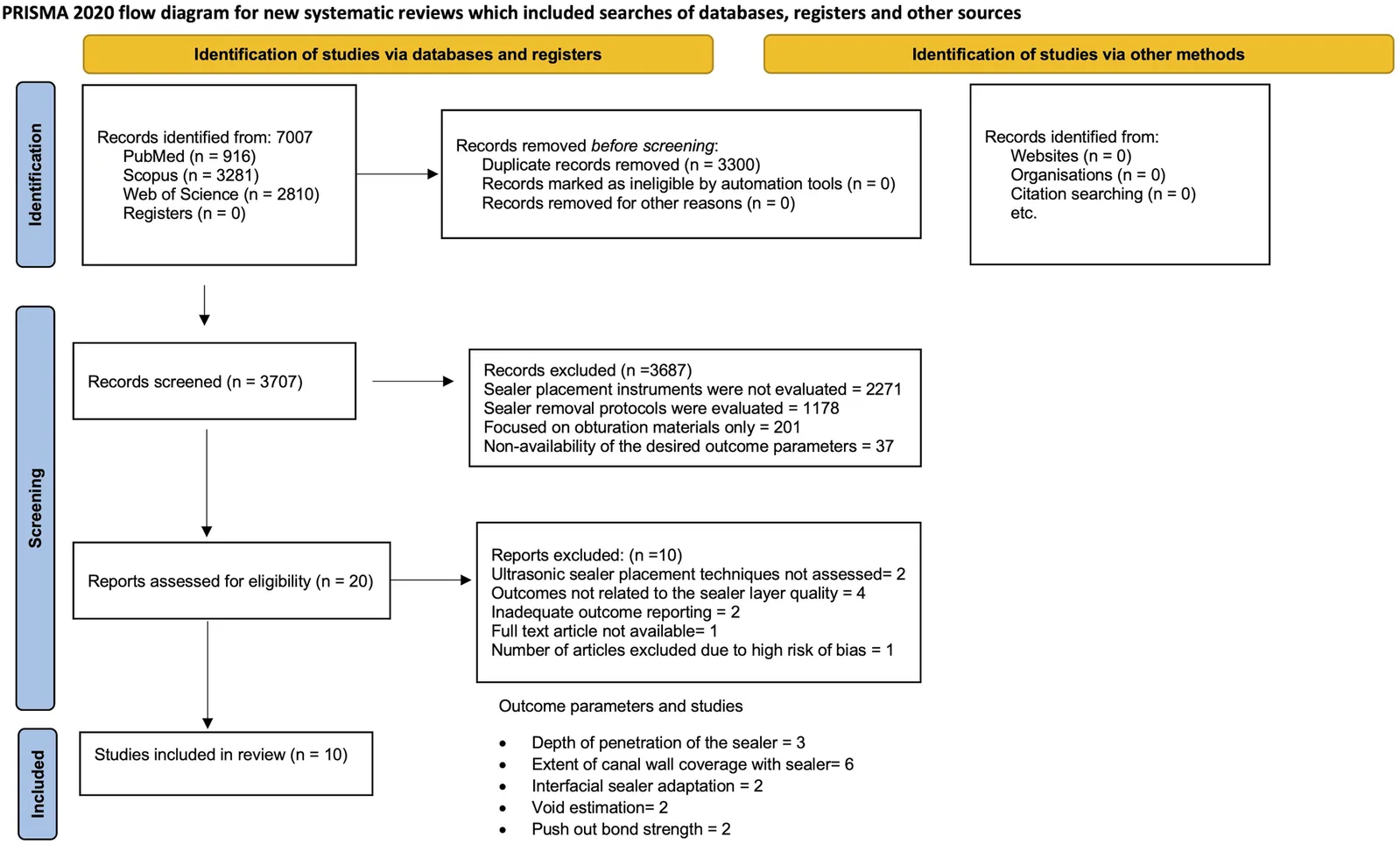
PRISMA 2020 flow diagram depicting the study screening process for the systematic review.

This systematic review was conducted and reported in accordance with the Preferred Reporting Items for Systematic Reviews and Meta-Analyses (PRISMA) 2020 guidelines (Figure 1).

The screening process was independently performed by two review authors (AB & NHK) based on the inclusion and exclusion criteria. Any discrepancies regarding study eligibility were resolved through discussion to reach consensus, with consultation of a third reviewer (MNH) when required. Specific search strings with key words such as “root canal obturation”, “endodontic sealer”, “Lentulo spiral”, “K file”, “ultrasonic”, “sonic”, “micro computed tomography”, “confocal laser scanning microscope”, “voids”, “depth of penetration”, “sealer penetration”, “marginal adaptation” and “push out bond strength” were used for literature search in databases such as PubMed, Scopus and Web of Science. A total of 7,007 articles [PubMed (*n* = 916), Scopus (*n* = 3,281), Web of Science (*n* = 2,810)] were retrieved through the initial database search (Supplementary File S1- Search Strategy). After the removal of 3,300 duplicate records, 3,707 articles were subjected to title and abstract screening. At this stage, 3,687 articles were removed: 2,271 did not evaluate sealer placement instruments, 1,178 focused exclusively on sealer removal protocols, 201 investigated obturation materials without assessing placement techniques, and 37 did not report the relevant outcome parameters. The remaining 20 articles underwent full-text screening, after which 9 studies were excluded. Two studies did not evaluate ultrasonic sealer placement, four did not assess outcomes related to sealer layer quality, two reported outcomes inadequately, and one study was excluded due to the unavailability of the full text.

### Assessment of risk of bias in included studies

**Figure 2:**
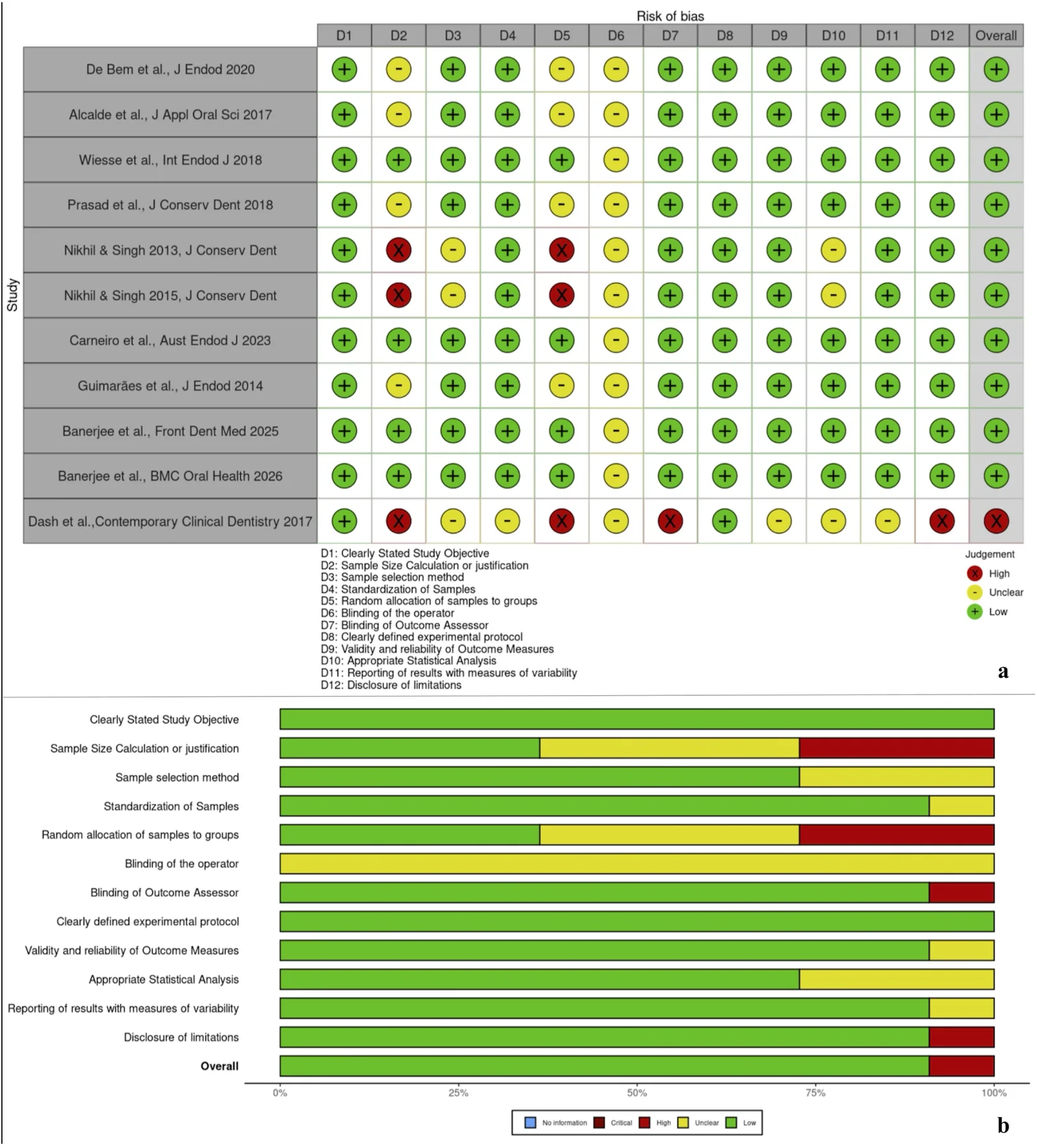
**(a)** traffic light plot illustrating the risk of bias assessment of the 11 included *in vitro* studies. Each row represents an individual study, while each column corresponds to a specific domain of bias assessment, including clarity of study objective, sample size calculation or justification, sample selection method, standardization of samples, random allocation of samples to groups, blinding of the operator, blinding of the outcome assessor, clarity of the experimental protocol, validity and reliability of outcome measures, appropriateness of statistical analysis, reporting of results with measures of variability, disclosure of limitations, and overall risk of bias. **(b)** Summary bar plot showing the proportion of studies classified as low, unclear, or high risk of bias across each evaluated domain and for the overall risk of bias assessment.

A risk of bias assessment was done using the Quality Assessment Tool for *in vitro* Studies (QUIN) (12). 12 domains were evaluated: clearly stated study objective, sample size calculation or justification, sample selection method, standardisation of samples, random allocation of samples to groups, blinding of operator, blinding of outcome assessor, clearly defined experimental protocol, validity and reliability of outcome measures, appropriate statistical analysis, reporting of results with measures of variability and disclosure of limitations. Each domain of the QUIN tool was scored on a three-point scale, with a score of 2 assigned when the criterion was adequately reported (low risk of bias), 1 when reporting was inadequate (unclear risk of bias), and 0 when the criterion was not reported (high risk of bias). The total methodological quality score for each study ranged from 0 to 24. Based on the percentage of the maximum attainable score, studies were categorised as having a low risk of bias when they achieved ≥70% of the total score, a moderate risk of bias when scores ranged between 50% and 69%, and a high risk of bias when scores were <50%. In the present systematic review, ten studies (8, 10, 11, 13–19) showed low risk of bias, while one showed high risk of bias (20), which was eliminated. Consequently, ten studies were included in the final qualitative synthesis (Figure 2, Supplementary File S2- Risk of Bias).

### Data extraction

The following data were extracted from the included studies: study design, sample size, tooth type, instrumentation system used, extent of apical preparation, irrigation protocol, type of sealer used, sealer mixing and dispensing protocols, instrument used for sealer placement, sealer placement method and mechanism of activation, canal region assessed, primary outcomes assessed, outcome assessment methods and key findings (Supplementary File S3- Data Extraction).

### Meta-analysis

A formal assessment of the eligibility of the included studies for quantitative meta-analysis was undertaken before data synthesis.

Among the ten studies included in the qualitative synthesis, 8 reported the necessary summary statistics (means and standard deviations) required for effect size computation. The remaining 2 studies reported results as medians with interquartile ranges or ranges only—a consequence of non-normal data distribution—and could not be pooled. These studies are discussed narratively alongside the pooled results.

The feasibility of pooling outcome data was evaluated based on three criteria: the number of studies reporting a given outcome using a comparable metric, the consistency of the outcome assessment methodology across studies, and the availability of the necessary summary statistics (means and standard deviations) to enable the computation of effect sizes.

Of the five outcome domains evaluated in this review—sealer penetration depth, percentage of canal wall coverage, push-out bond strength, interfacial adaptation, and void volume—only two were considered eligible for quantitative pooling. The percentage of canal wall coverage with sealer was the most commonly reported outcome, which was assessed in six out of the eight studies considered for meta-analysis. Sealer penetration depth was assessed in three of the included studies using confocal laser scanning microscopy (CLSM) with quantitative reporting of mean penetration depth in micrometres. Push-out bond strength was reported in two studies using standardised mechanical testing, with results expressed in megapascals (MPa). Interfacial adaptation was reported in two studies; however, heterogeneity in the metric definitions and scoring scales used across studies precluded meaningful pooling. Void volume was assessed in only two studies using different imaging modalities (CLSM and micro-computed tomography), rendering quantitative synthesis inappropriate for this outcome.

Hence, among the 8 studies assessed for quantitative synthesis, 6 provided comparable and extractable data for the outcomes sealer penetration depth and percentage of canal wall coverage and were therefore included in the final meta-analysis. The remaining 2 studies primarily reporting push-out bond strength and void-related outcomes were included only in the narrative synthesis.

For the eligible outcomes, a random-effects meta-analysis model was planned, given the anticipated clinical and methodological heterogeneity across the included *in vitro* studies. The weighted mean difference (WMD) with 95% confidence intervals was selected as the primary effect measure, as the eligible outcomes were measured on a common scale across studies. Statistical heterogeneity was to be assessed using the *I*^2^ statistic, with values of 25%, 50%, and 75% interpreted as representing low, moderate, and substantial heterogeneity, respectively. The significance of heterogeneity was to be assessed using the Chi^2^ test, with a threshold of *p* < 0.10. In view of the extremely high heterogeneity anticipated across these *in vitro* comparisons, 95% prediction intervals were also planned for the pooled outcomes, in addition to 95% confidence intervals, to convey the expected range of true effects across differing study conditions.

Given the recognised sources of clinical heterogeneity among the included studies, pre-specified subgroup analyses were planned for both eligible outcomes. The primary subgrouping variable was sealer class, distinguishing between epoxy resin-based sealers (including AH Plus, AH 26, Acroseal, Adseal, EndoREZ, and Sealer 26) and calcium silicate-based sealers (including EndoSequence BC Sealer, Sealer Plus BC, Bio-C Sealer, and MTA Fillapex). This distinction was considered clinically important, as the rheological and physicochemical properties of these sealer classes differ substantially and may influence the extent of response to ultrasonic activation. A secondary subgroup analysis by canal level (coronal, middle, and apical thirds) was planned where study-level data permitted, to examine whether the effect of ultrasonic activation on sealer penetration depth varied across root canal regions. Additionally, where data were available, a subgroup analysis by comparator technique (Lentulo spiral, hand file, sonic activation) was to be performed to explore whether the comparative effectiveness of ultrasonic placement differed depending on the non-ultrasonic technique evaluated.

Publication bias was not formally assessed given that the number of included studies was below the recommended threshold of ten for funnel plot interpretation. Funnel plots were nonetheless generated for descriptive purposes only; any visual asymmetry observed was not used to draw formal conclusions regarding the presence of publication or reporting bias, consistent with the limited interpretability of funnel plots when fewer than ten studies contribute data. As all included studies attained a low risk-of-bias score on the QUIN tool, sensitivity analyses were not performed based on methodological quality. Two of the included studies were conducted by authors of the present review (11, 19). Among these, only Banerjee et al. (19) contributed data to the pooled outcomes, whereas Banerjee et al. (11) assessed void volume by micro-computed tomography and was included in the narrative synthesis only. To assess the influence of author overlap on the pooled estimates, a sensitivity analysis was therefore performed for both pooled outcomes with Banerjee et al. (19) excluded, and the resulting weighted mean differences, 95% confidence intervals, *I*^2^ values and prediction intervals were compared with the primary analyses.

All meta-analytic computations were performed using R version 4.5.0 (R Foundation for Statistical Computing, Vienna, Austria), using the metafor package (version 4.8-0). Forest plots and a funnel plot were generated using ggplot2.

Since several studies contributed multiple comparisons (for example, different canal levels, sealers, or comparator techniques), the observed effect sizes were not independent. A three-level random-effects model was therefore fitted using the rma.mv() function in metafor, in which Level 1 represents the sampling variance of each observed comparison, Level 2 represents variation between comparisons nested within the same study, and Level 3 represents variation between studies. Variance components at Levels 2 and 3 were estimated by restricted maximum likelihood (REML). The necessity of the nested structure was examined using likelihood-ratio tests comparing the full model with reduced models in which each variance component was constrained to zero. Total *I*^2^ was calculated using the multilevel formulation of Cheung (21) and was additionally decomposed to quantify the proportion of variability attributable to within-study and between-study sources.

Three further analyses were performed to examine the robustness of the pooled estimates to the dependency structure. First, because some studies compared a single ultrasonic arm against more than one comparator, the exact covariance induced by these shared arms was incorporated into the variance-covariance matrix and the models re-fitted. Second, the analyses were repeated retaining only one comparison per ultrasonic arm. Third, cluster-robust variance estimation (CR2, clustered by study) was applied to obtain standard errors that do not depend on the assumed variance structure being correctly specified.

Certainty of evidence was not rated using the GRADE approach. GRADE is designed to rate confidence in estimates of effect for patient-important outcomes in clinical studies, and the GRADE Working Group has not issued guidance for the appraisal of *in vitro* research. As all outcomes evaluated in this review are laboratory surrogate measures obtained from extracted teeth, and as no defined starting certainty exists for such designs, formal GRADE ratings would convey a false impression of comparability with clinical evidence syntheses. Instead, a structured narrative appraisal was undertaken using the GRADE domains of risk of bias, inconsistency, indirectness, imprecision and publication bias, adapted to the laboratory context and reported without assignment of formal certainty ratings (Table 1).

**Table 1:** Adapted from the GRADE domains for the appraisal of *in vitro* evidence.

Domain	Judgement	Basis
Risk of bias	Not serious	All ten included studies rated low risk of bias on the QUIN tool.
Inconsistency	Very serious	*I*^2^ 99.0–100.0%; *τ* large relative to the pooled effect; the coverage prediction interval crosses the null.
Indirectness	Very serious	Surrogate laboratory outcomes on extracted teeth; no patient-important outcome; the link to clinical success is unestablished.
Imprecision	Serious	Only 3–4 studies per outcome; cluster-robust intervals are far wider than model-based intervals.
Publication bias	Undetectable	Fewer than ten studies; funnel-plot asymmetry cannot be distinguished from heterogeneity.
Overall	Very low confidence	Findings are exploratory and hypothesis-generating; they do not support clinical recommendations.

Formal GRADE certainty ratings were not assigned, as GRADE is designed for clinical studies reporting patient-important outcomes. Judgements are reported descriptively and should not be interpreted as equivalent to GRADE certainty levels.

The numerical data extracted from the studies and used in the meta-analysis are provided in Supplementary File S4- Data for Meta-analysis.

The raw data and R code used for the meta-analysis have been deposited in Figshare and are publicly available at: doi: 10.6084/m9.figshare.32124760.

## Results

### Study selection

The study selection process, conducted as per the PRISMA guidelines, yielded a total of 11 articles. Among these articles, one study by Dash et al. (20) was excluded due to high risk of bias. The remaining 10 articles were included in the qualitative synthesis and considered for quantitative data synthesis where appropriate. The included studies investigated the influence of sealer placement techniques on the quality of the sealer layer formed within instrumented root canals.

### General characteristics of included studies

All the studies followed an *in vitro* study design using intact human teeth extracted for orthodontic or periodontal purposes. The total sample size varied from 30 to 100 per study.

The included studies under assessment predominantly utilised teeth with single straight canals to reduce the chances of anatomical variability. One study used permanent maxillary central incisors (10), two studies used canine teeth (16, 17), two studies included mandibular premolars (11, 19), two studies evaluated specific roots of molar teeth (13, 14), and three studies investigated single-rooted teeth without specifying the tooth type (8, 15, 18). All the studies followed strict inclusion criteria at the time of sample selection, wherein the specimens were required to have fully formed apices and be devoid of caries, cracks or fractures, calcifications, or previous endodontic treatment.

The biomechanical preparation of the canals was performed using Nickel-Titanium rotary instruments in all included studies, although the specific file systems and apical preparation sizes varied. NaOCl and EDTA were used as standard irrigating solutions in all the included studies.

### Sealer types and placement techniques

A variety of root canal sealers were used across the studies, such as Epoxy resin-based and Calcium silicate-based sealers. Manufacturer instructions were followed in the dispensing and manipulation of the sealer in all the studies. The primary focus of all studies was the method of sealer placement, with ultrasonic activation evaluated in comparison with non-ultrasonic techniques. Comparator techniques included manual placement using hand files, rotary placement using Lentulo spirals or rotary files, and sonic-activated instruments. Ultrasonic sealer agitation was performed using piezoelectrically activated tips, files, NiTi finger spreader, or novel vibratory instruments such as the Vibrospiral.

### Outcome measures and assessment methods

Sealer layer quality was assessed using a range of outcome measures. The most frequently evaluated outcomes were extent of canal wall coverage with sealer and its penetration depth. Additional parameters evaluated included void formation in the sealer layer, interfacial adaptation of the sealer with root canal dentin, and push-out bond strength. Outcome assessment methods varied across studies. Confocal laser scanning microscopy (CLSM) was predominantly used to evaluate percentage of canal coverage, sealer penetration depth, and interfacial adaptation. Micro–computed tomography was employed to assess void volume, and mechanical testing, such as the push-out test, was used for bond strength evaluation. The outcomes were assessed at multiple canal levels, typically the coronal, middle, and apical thirds, for all the included studies.

Across the included studies, ultrasonic sealer placement was consistently associated with more favourable outcomes with respect to the quality of the sealer layer formed within the root canal system. In studies evaluating sealer distribution by confocal laser scanning microscopy (CLSM), ultrasonic activation was associated with greater depth of sealer penetration, higher percentage of canal wall coverage, and improved interfacial adaptation (8, 10, 13–16, 18, 19). Studies assessing voids using CLSM (16) and micro-computed tomography (micro-CT) (11) similarly reported a more homogeneous sealer layer with fewer voids following ultrasonic placement. In addition, studies evaluating dislodgement resistance using the push-out bond strength test (13, 15, 17) showed more favourable bond strength values with ultrasonic sealer placement. Hence, the available *in vitro* evidence suggests that ultrasonic-assisted placement was associated with more favourable sealer layer characteristics across the outcome domains assessed, although these laboratory findings should not be extrapolated as evidence of clinical superiority.

### Quantitative synthesis (meta-analysis)

#### Outcome 1: sealer penetration depth (µm)

**Figure 3:**
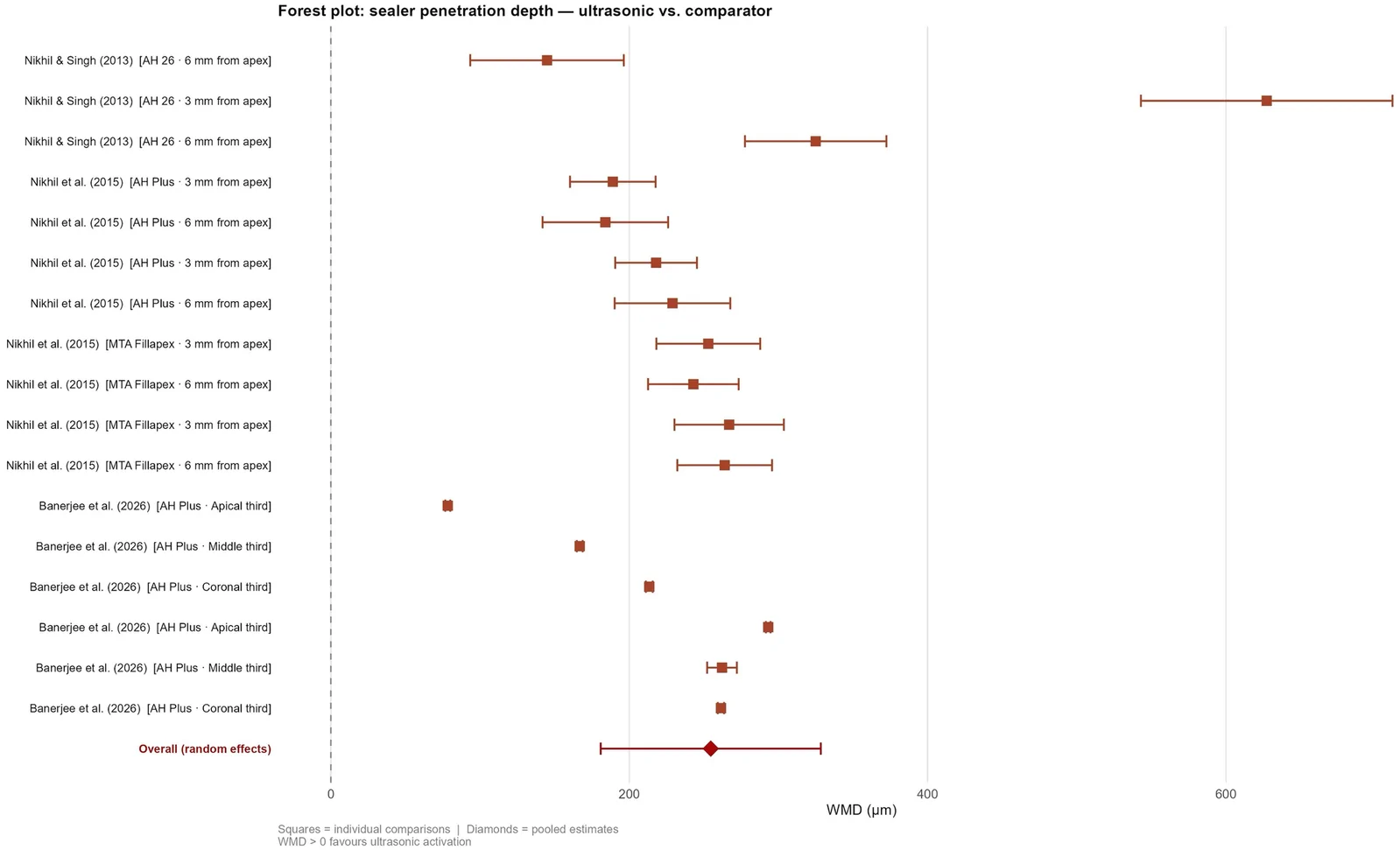
Forest plot comparing sealer penetration depth (µm) between ultrasonic activation and comparator techniques. 95% prediction interval: 28.54–480.77 µm. Weighted mean difference (WMD) values greater than 0 favour ultrasonic activation.

Three studies [(8, 10, 19); *k* = 17 comparisons) were eligible for pooling of sealer penetration depth. The three-level random-effects model yielded a pooled WMD of 254.66 µm (95% CI: 180.88 to 328.44; *p* < 0.001) in favour of ultrasonic activation (Figure 3). Total heterogeneity was very high (*I*^2^ = 100.0%), with variance components of *τ*^2^ = 9,558.60 at the within-study level and *τ*^2^ = 2,333.77 at the between-study level, corresponding to a total *τ* of 109.05 µm. Decomposition indicated that 80.4% of total variability arose from differences between comparisons within the same study and 19.6% from differences between studies, with sampling error contributing negligibly. The corresponding 95% prediction interval was 28.54 to 480.77 µm; although wide, it remained entirely above the line of no effect, indicating that a comparable future study would still be expected to favour ultrasonic activation, albeit with considerable uncertainty as to the magnitude of the difference.

**Figure 4:**
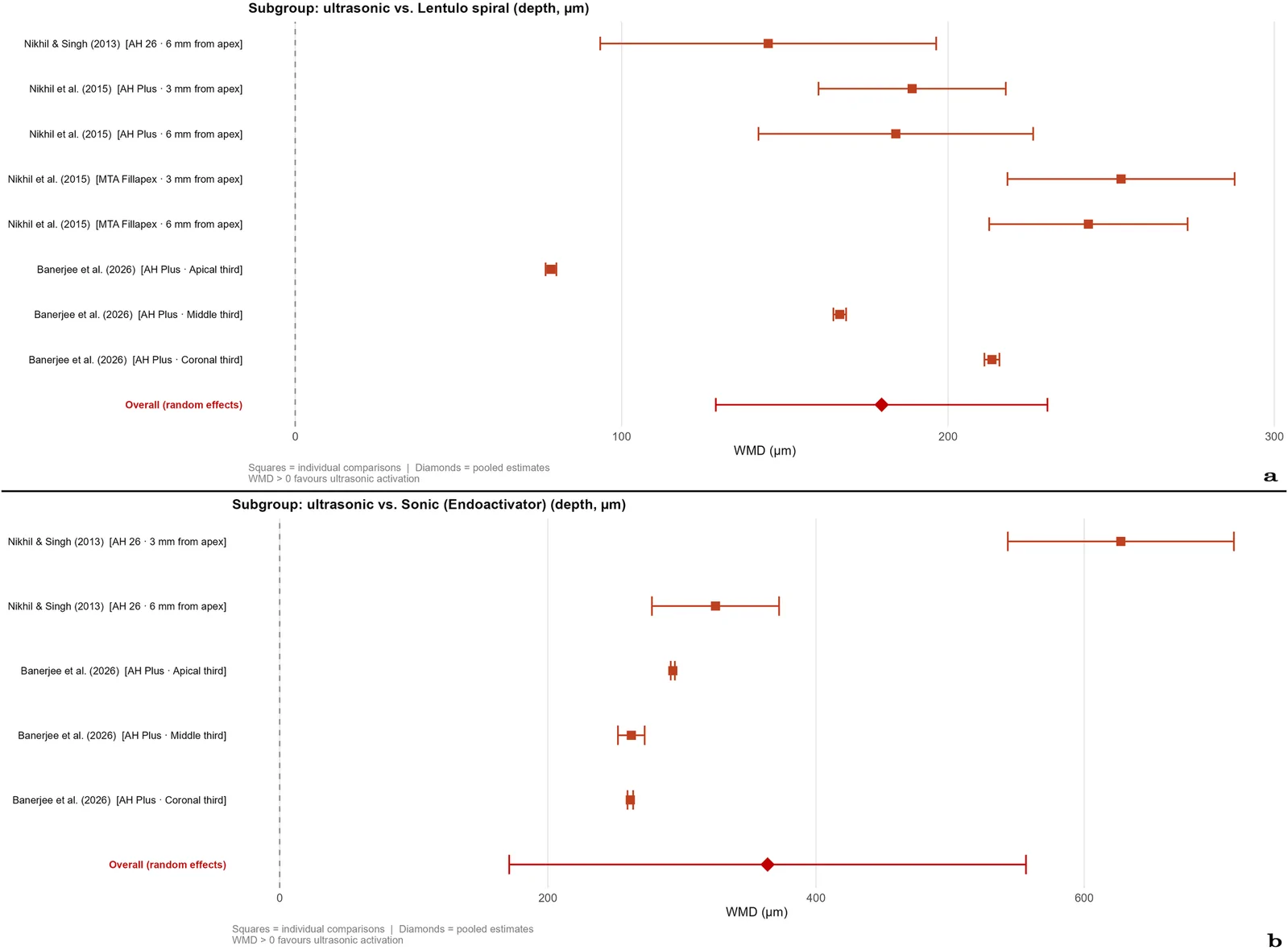
**(a)** subgroup forest plot comparing sealer penetration depth (µm) between ultrasonic activation and Lentulo spiral placement. Weighted mean difference (WMD) values greater than 0 favour ultrasonic activation. **(b)** Subgroup forest plot comparing sealer penetration depth (µm) between ultrasonic activation and Endoactivator placement. Weighted mean difference (WMD) values greater than 0 favour ultrasonic activation.

Pre-specified subgroup analyses by comparator technique demonstrated that the advantage of ultrasonic activation was consistently observed irrespective of the comparator used. When compared specifically with Lentulo spiral placement (*k* = 8, *n* = 3 studies), the pooled WMD was 179.65 µm (95% CI: 128.86 to 230.45; *p* < 0.001; *I*^2^ = 99.9%), and when compared with sonic (EndoActivator) placement (*k* = 5, *n* = 2 studies), the pooled WMD was 363.85 µm (95% CI: 171.14 to 556.57; *p* = 0.0002; *I*^2^ = 100.0%) (Figure 4). Subgroup analysis by sealer class for epoxy resin-based sealers (*k* = 13, *n* = 3 studies) yielded a pooled WMD of 248.86 µm (95% CI: 162.51 to 335.22; *p* < 0.001; *I*^2^ = 100.0%). Calcium silicate-based sealers were represented by a single study in this outcome domain and were therefore not pooled separately. Canal-level subgroup analyses were feasible for the 3 mm from apex (*k* = 5; WMD = 425.64 µm; 95% CI: 36.65 to 814.64; *p* = 0.032) and 6 mm from apex (*k* = 6; WMD = 232.41 µm; 95% CI: 184.25 to 280.56; *p* < 0.001) levels, both consistently favouring ultrasonic activation, though with considerably wider confidence intervals at the apical level reflecting greater variability.

#### Outcome 2: percentage of canal wall coverage with sealer (%)

**Figure 5:**
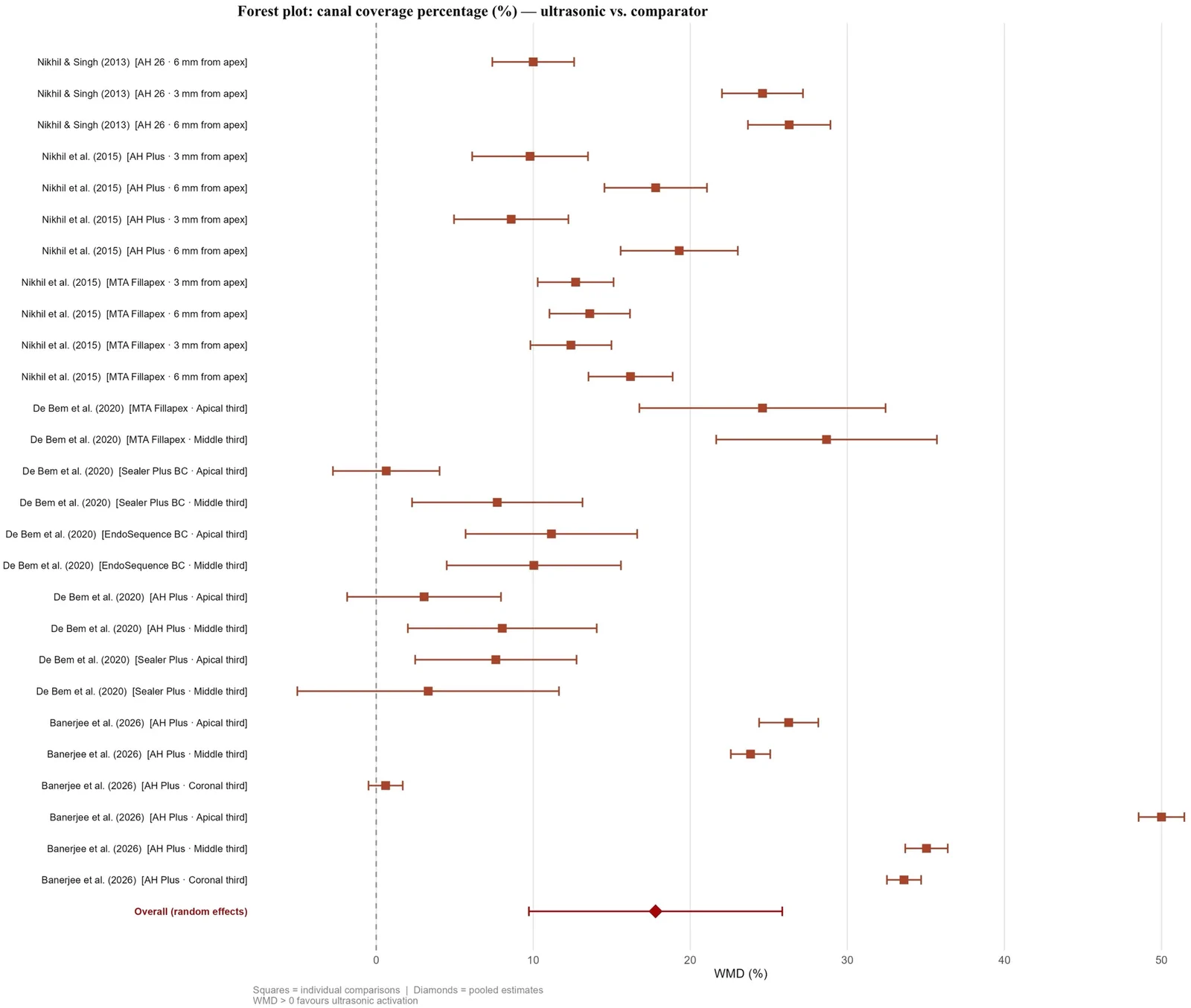
Forest plot comparing the percentage of canal wall coverage with sealer (%), comparing ultrasonic activation with comparator techniques. 95% prediction interval: −7.45–43.04%. Weighted mean difference (WMD) values greater than 0 favour ultrasonic activation.

**Figure 6:**
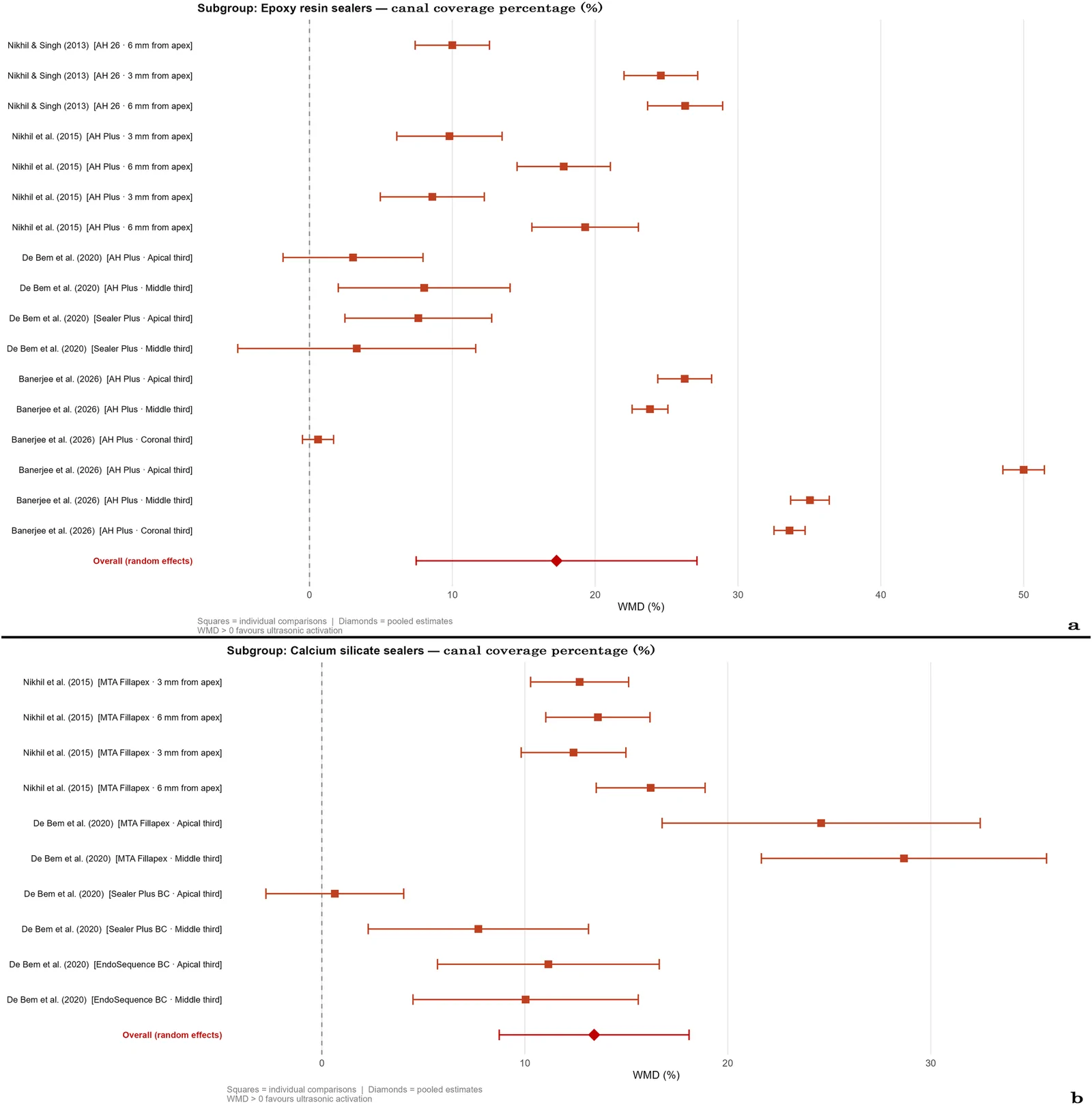
**(a)** forest plot of percentage of canal wall coverage with sealer (%) for epoxy resin-based sealers. Weighted mean difference (WMD) values greater than 0 favour ultrasonic activation. **(b)** Forest plot of percentage of canal wall coverage with sealer (%) for calcium silicate-based sealers. Weighted mean difference (WMD) values greater than 0 favour ultrasonic activation.

Six studies reported the percentage of canal wall coverage with sealer (8, 10, 14–16, 19); however, four studies (*k* = 27 comparisons) provided sufficiently comparable quantitative data and were included in the meta-analysis. Studies by Nikhil & Singh (8), Nikhil et al. (10), De Bem et al. (15), and Banerjee et al. (19); *k* = 27 comparisons were pooled for the percentage of canal wall coverage. The overall pooled WMD was 17.79% (95% CI: 9.73 to 25.86; *p* < 0.001; *I*^2^ = 99.1%) in favour of ultrasonic placement (Figure 5). Total heterogeneity was high (*I*^2^ = 99.1%), with variance components of *τ*^2^ = 98.50 within studies and *τ*^2^ = 50.48 between studies (total *τ* = 12.21%); 65.5% of total variability was attributable to within-study differences and 33.6% to between-study differences. The 95% prediction interval was −7.45% to 43.04%, crossing the null and indicating that a negligible or reversed effect cannot be excluded under some study conditions, and underscoring that the pooled estimate should be read as an exploratory summary across heterogeneous conditions. Subgroup analysis by sealer class demonstrated a consistent benefit for both epoxy resin-based sealers (*k* = 17, *n* = 4 studies; WMD = 17.30%; 95% CI: 7.47 to 27.13; *p* = 0.0006; *I*^2^ = 99.5%) (Figure 6A) and calcium silicate-based sealers (*k* = 10, *n* = 2 studies; WMD = 13.42%; 95% CI: 8.74 to 18.09; *p* < 0.001; *I*^2^ = 93.9%) (Figure 6B). Heterogeneity remained high for both subgroups, reflecting variability in sealers assessed and canal levels evaluated across studies.

Likelihood-ratio tests supported retention of the within-study variance component for both outcomes (penetration depth, LRT = 39,391.33, *p* < 0.001; canal-wall coverage, LRT = 3,603.25, *p* < 0.001). The between-study component was supported for canal-wall coverage (LRT = 4.26, *p* = 0.039) but not for penetration depth (LRT = 0.30, *p* = 0.585), consistent with the small number of contributing studies. Moderator analyses indicated that the comparator technique accounted for a substantial proportion of the observed variability, explaining 36.8% of the variance for penetration depth (Qₘ = 14.03, df = 2, *p* = 0.0009) and 51.3% for canal-wall coverage (Qₘ = 21.04, df = 2, *p* < 0.001). In contrast, neither canal level (*p* = 0.651 and *p* = 0.477) nor sealer class (*p* = 0.712) explained any appreciable proportion of the variance.

Incorporating the covariance induced by shared ultrasonic arms altered the pooled estimates negligibly (penetration depth 255.51 µm, 95% CI: 179.93 to 331.08; canal-wall coverage 17.80%, 95% CI: 9.72 to 25.87). Retaining only a single comparison per ultrasonic arm likewise preserved both effects (penetration depth 233.72 µm, 95% CI: 124.47 to 342.98; canal-wall coverage 12.85%, 95% CI: 8.84 to 16.86). Cluster-robust variance estimation produced considerably wider intervals (penetration depth 254.66 µm, 95% CI: 90.62 to 418.69, *p* = 0.022; canal-wall coverage 17.79%, 95% CI: 4.51 to 31.08, *p* = 0.024), although with only three to four clusters these robust estimates are themselves imprecise and are reported as an indication that the model-based intervals should not be over-interpreted.

#### Outcome 3: push-out bond strength (MPa)

Push-out bond strength data meeting eligibility criteria for pooling were available from a single study [Wiesse et al. (17); *k* = 10 comparisons]. Cross-study meta-analysis was therefore not feasible, and a within-study summary was computed separately by sealer type. For AH Plus sealer, ultrasonic activation yielded a mean difference of 0.95 MPa (95% CI: 0.45 to 1.46; *p* = 0.0002) in favour of ultrasonic placement across four canal-level comparisons. For MTA Fillapex sealer, the corresponding mean difference was 0.52 MPa (95% CI: 0.18 to 0.87; *p* = 0.0032) across six comparisons. These results indicate consistently higher bond strength following ultrasonic activation for both sealer types, though they should be interpreted as single-study estimates rather than cross-study meta-analytic pooled values. De Bem et al. (15), who also assessed bond strength, reported results as medians with interquartile ranges and could not be pooled.

#### Publication bias

**Figure 7:**
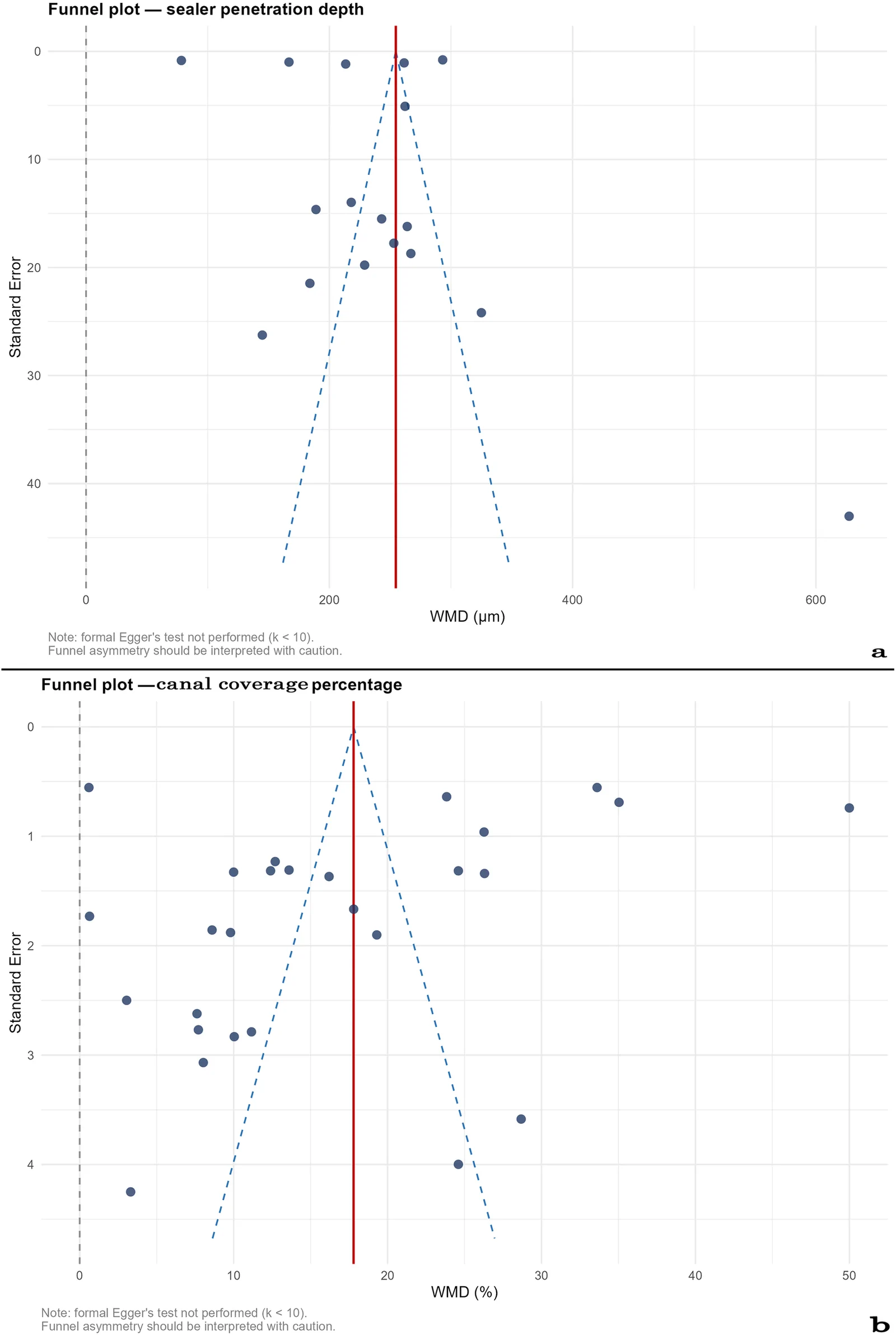
Funnel plot (descriptive only). **(a)** Sealer penetration depth (WMD, µm). **(b)** canal coverage percentage (WMD, %). Formal assessment of publication bias using Egger's test was not performed because fewer than 10 studies were included for meta-analysis.

Funnel plots were generated for sealer penetration depth (Figure 7A) and percentage of canal-wall coverage (Figure 7B). As the number of studies contributing comparisons was below ten, formal testing for funnel-plot asymmetry (e.g., Egger's test) was not performed and the plots are presented for descriptive purposes only. Any visual asymmetry with fewer than ten studies cannot reliably be distinguished from that expected under high heterogeneity or small-study effects, and no inference regarding publication or reporting bias has been drawn from these plots.

#### Sensitivity analysis

Since Banerjee et al. (19) was conducted by the authors of the present review and was the only self-authored study contributing to the pooled outcomes, each analysis was re-estimated with that study excluded. For sealer penetration depth (11 comparisons from 2 studies), the pooled WMD was 283.66 µm (95% CI: 160.98 to 406.35; *p* < 0.001; *I*^2^ = 98.0%); for percentage of canal-wall coverage (21 comparisons from 3 studies), the pooled WMD was 13.99% (95% CI: 8.80 to 19.18; *p* < 0.001; *I*^2^ = 94.7%). In both cases, the direction and statistical significance of the pooled effect were preserved, and the magnitude remained broadly consistent with the primary analysis, indicating that author overlap did not materially alter the findings. Given the small number of remaining studies—particularly for penetration depth, where only two studies contributed—these re-estimated values should be regarded as a robustness check rather than independent confirmatory estimates (Table 2).

**Table 2:** Sensitivity analysis excluding the self-authored study.

Outcome	Analysis	*k* (studies)	WMD (95% CI)	*I* ^2^	95% prediction interval
Penetration depth (µm)	Primary	17 (3)	254.66 (180.88, 328.44)	100.0%	28.54 to 480.77
Penetration depth (µm)	Sensitivity—Banerjee et al. (19) excluded	11 (2)	283.66 (160.98, 406.35)	98.0%	1.10 to 566.23
Canal-wall coverage (%)	Primary	27 (4)	17.79 (9.73, 25.86)	99.1%	−7.45 to 43.04
Canal-wall coverage (%)	Sensitivity—Banerjee et al. (19) excluded	21 (3)	13.99 (8.80, 19.18)	94.7%	−1.84 to 29.82

All models are two-level random-effects (REML) via rma.mv(); WMD > 0 favours ultrasonic activation; all *p* < 0.001.

Primary and sensitivity meta-analyses of sealer penetration depth and canal-wall coverage. Sensitivity analyses excluding the self-authored study (19) demonstrated that the primary findings remained robust. Positive WMD values favour ultrasonic activation.

## Discussion

The present systematic review aims to assess the influence of ultrasonic and non-ultrasonic sealer placement techniques on the quality of the endodontic sealer layer.

Endodontic sealers play a pivotal role in the process of obturation. They form a thin intermediary layer between the gutta-percha and the root canal walls, filling the residual spaces between them, thereby contributing to the establishment of a fluid-impervious three-dimensional seal (22, 23). This, in turn, prevents microleakage and the ingress of microorganisms, which contributes to a successful endodontic treatment (24). An ideal sealer layer is characterised by minimal thickness, effective intratubular penetration, complete coverage of the canal walls, intimate interfacial adaptation, and absence of voids (6). Since the method of sealer placement directly influences these characteristics, it has an important bearing on the overall quality of obturation (8).

A wide range of sealer placement techniques has been investigated in the literature, including manual methods such as K-files, rotary techniques such as Lentulo spirals and Endomotor-driven endodontic files, and activation-based approaches employing ultrasonic and sonic devices. Despite the growing body of evidence, there is no clear consensus in the existing literature regarding the optimal method of sealer placement. The findings of the present review indicate that ultrasonic-assisted sealer placement was consistently associated with more favourable sealer layer characteristics, including greater depth of sealer penetration, improved canal wall coverage, enhanced interfacial adaptation, fewer voids, and higher push-out bond strength when compared with manual, rotary, and sonic techniques; however, these findings are derived exclusively from laboratory data and should be interpreted with corresponding caution.

### CLSM-based assessment of sealer layer characteristics: depth of penetration, canal wall coverage, interfacial adaptation

Among the included studies, confocal laser scanning microscopy (CLSM) was the most-used method for evaluating sealer layer quality, particularly for depth of sealer penetration, percentage of canal wall coverage, and interfacial adaptation. Nikhil et al. (8), Guimarães et al. (16), Nikhil et al. (10), Alcalde et al. (14), Wiesse et al. (17), Prasad et al. (18), De Bem et al. (15), Carneiro et al. (13), and Banerjee et al. (19) employed CLSM to assess these parameters and collectively reported more favourable sealer layer characteristics with ultrasonic-assisted placement compared with comparator techniques such as Lentulo spirals, Endoactivator, and rotary endodontic files.

Sealer penetration into dentinal tubules is influenced by multiple factors, including the removal of the smear layer, tubular density and diameter, root canal anatomy, moisture presence, and the sealer's physical and chemical characteristics (22, 25–27). Across the included studies, these variables were generally controlled by standardising tooth type, instrumentation protocol, sealer type and obturation protocol within each experimental design, thereby reducing the influence of confounding factors on the assessment of the various sealer placement techniques.

The widespread use of CLSM across the included studies for evaluating sealer layer characteristics may be attributed to its ability to provide high-resolution, high-contrast visualisation of the sealer–dentine interface when fluorescent dyes such as Rhodamine B are incorporated into the sealer. Bright fluorescent sealer tags within the dentinal tubules and circumferential fluorescent rings along the canal wall can be visualised clearly even when present sparsely. Hence, CLSM enables a more accurate assessment of intratubular sealer penetration and canal wall coverage than other techniques, such as scanning electron microscopy (SEM) (28–30). In addition, CLSM permits evaluation of specimens in ambient air without the need for extensive chemical preparation, thereby reducing the likelihood of artefacts and interface distortion (31). Hence, CLSM appears to be an appropriate and reliable method for evaluating sealer penetration, canal wall coverage, and sealer adaptation to dentinal walls.

Nikhil and Singh (8) compared an ultrasonically activated tapered file with Lentulo spiral and Endoactivator as sealer coating devices. CLSM visualisation of the sealer layer indicated that the ultrasonic method produced the greatest depth of sealer penetration and canal wall coverage, suggesting that acoustic agitation improved sealer distribution more effectively than either rotary or sonic placement.

A similar trend was observed in studies by Guimarães et al. (16), Alcalde et al. (14), Prasad et al. (18), De Bem et al. (15), and Carneiro et al. (13), all of which compared sealer placement using a Lentulo spiral alone with Lentulo placement followed by ultrasonic agitation. For ultrasonic activation, Guimarães et al. used a NiTi finger spreader attached to an ultrasonic device, Carneiro et al. used an E1 ultrasonic tip, Alcalde et al. and De Bem et al. used Irrisonic tips, while Prasad et al. did not specify the type of ultrasonic tip used. Across these studies, the addition of ultrasonic agitation consistently improved dentinal sealer penetration and reduced interfacial gaps, highlighting the benefit of secondary ultrasonic activation even when the sealer is initially placed using a conventional carrier.

Nikhil et al. (10), compared an ultrasonic endodontic tip (K15 Sonofile), with two types of rotary sealer placement devices, namely, a Lentulo spiral, and a rotary ProTaper F1 file attached to an Endomotor. The ultrasonic group exhibited more favourable sealer layer characteristics, reinforcing the suggestion that piezoelectric agitation of sealer may lead to better sealer distribution than rotational modes of sealer placement, irrespective of the design of the rotary device.

Wiesse et al. (17) and Banerjee et al. (19) compared ultrasonic coating of sealer with sonic placement. For the piezoelectric group, Wiesse et al. used an ultrasonic tip, while Banerjee et al. used the novel sealer-placing instrument, Vibrospiral. Moreover, Wiesse et al. used an additional non-activated control group, while Banerjee et al. used Lentulo spiral placement as the conventional comparator. Despite the differences in study design, both investigations consistently reported more favourable sealer layer characteristics featuring minimal thickness, uniformity, and adaptation to canal walls with the ultrasonic mode of placement than with sonic agitation or the use of a Lentulo spiral. A key development in the study by Banerjee et al. was the introduction of the novel piezoelectric sealer coating instrument, Vibrospiral. Unlike protocols in which ultrasonic agitation is performed after sealer delivery, the Vibrospiral functioned as both a carrier and an activation instrument, allowing simultaneous placement and ultrasonic agitation.

Despite the methodological variations among the included studies, ultrasonic activation consistently resulted in greater intratubular penetration, intimate adaptation to canal walls, and more uniform circumferential sealer distribution than Lentulo spirals, hand files, and non-activated placement techniques. This suggests that the principal advantage may not lie merely in the delivery instrument itself, but in the piezoelectric agitation imparted to the sealer.

### Push-out bond strength

Apart from the sealer layer assessment under CLSM, De Bem et al. (15), Carneiro et al. (13), and Wiesse et al. (17), further conducted the push-out bond strength test for all the groups. All the studies consistently reported significantly higher bond strength values following ultrasonic activation compared with manual and sonic techniques. The observed improvement in bond strength may be explained by greater sealer penetration into dentinal tubules in the ultrasonic group, which improved the micromechanical interlocking, resulting in enhanced resistance to dislodgement forces (32).

### Assessment of voids incorporated in the sealer layer using stereomicroscope and micro-CT

Void formation within the sealer layer is an important indicator of obturation quality, as the presence of internal or interfacial voids may compromise the continuity of the seal and create potential pathways for microleakage. In the present review, void-related outcomes were assessed in a limited number of studies using both stereomicroscope and micro-computed tomography (micro-CT).

Guimaraes et al. (16) used a stereomicroscope for the assessment of voids in the sealer layer and found no significant difference in the void percentage among the ultrasonically activated and non-activated groups.

Unlike the findings by Guimaraes et al., the Micro CT assessment of voids in the sealer layer by Banerjee et al. (11) depicted significantly reduced void volume percentage for ultrasonically active Vibrospiral in comparison to manual placement using K file and motorised rotational placement with Lentulo spiral. This may be attributed to the fact that a stereomicroscope is less sensitive, while micro–computed tomography allows accurate, non-destructive assessment of root canal morphology by generating high-resolution datasets that can be reconstructed in multiple planes and visualised as two- or three-dimensional images. This technique permits independent or simultaneous evaluation of internal and external canal anatomy, thereby assisting in the assessment of voids in the sealer layer (33).

### Mechanistic comparison of ultrasonic and non-ultrasonic sealer placement techniques

The more favourable sealer layer characteristics consistently observed in the piezoelectrically activated groups across the included studies have been attributed to the acoustic microstreaming of the sealer produced by the ultrasonic sealer placement device. Piezoelectric energy generates multiple nodes and internodes along the length of the instrument, generating strong currents (34), which in turn deliver energy to the sealer (35). The ultrasonic energy increases the pressure of the sealer against the canal walls, which enables effective sealing of irregularities (16), accessory canals (36), and dentinal tubules, resulting in the formation of a greater number of longer, denser sealer tags. Moreover, the heat generated by piezoelectricity is optimal to enhance sealer fluidity, which also contributes to greater sealer penetration (37, 38).

This mechanism is particularly well illustrated by Vibrospiral, the novel ultrasonically activated sealer placement instrument evaluated by Banerjee et al. (11, 19), which combines sealer delivery and piezoelectric activation within a single device. Moreover, it has a flexible spring-like design that enables easy insertion of the instrument into tortuous canals, unlike rigid ultrasonic tips. Vibrospiral appears to optimise both sealer placement and distribution, producing a more homogeneous layer with deep penetration and fewer voids, in comparison to K file, Lentulo spiral and Endoactivator (11, 16, 19, 36).

Studies consistently demonstrated less favourable sealer layer characteristics when sonic activation devices such as the Endoactivator were used. In contrast to ultrasonic energy, sonic activation generates only a single node and antinode along the vibrating instrument, resulting in lower streaming velocity (8). In addition, sonic systems operate at a lower frequency and higher amplitude than ultrasonics, which may disturb the sealer layer and cause abrupt displacement during placement. This may promote air entrapment as the sealer sets, resulting in a more non-uniform and discontinuous sealer layer (37, 38). Although sonic activation is effective in producing hydrodynamic agitation in irrigation solutions (39), a similar effect does not appear to occur with root canal sealers, likely owing to their greater density and viscosity, which may limit the efficiency of sonic energy transfer (40). Consequently, sonic activation appears to provide less effective sealer adaptation to dentinal walls and reduced intratubular penetration when compared with ultrasonic activation (8).

The rotary motion of the Lentulo spiral results in a centrifugal action, depositing sealer on the canal walls. However, the high-speed rotary motion of the Lentulo spiral incorporates air bubbles in the sealer, leading to a voided, non-uniform sealer layer (41) and reduced intratubular sealer penetration (13).

Mechanised agitation of the sealer is absent in manual sealer placement using K-files. Hence, this method was associated with increased void formation within the sealer layer, as seen in the study by Banerjee et al. (11). Similar findings were reported by Kahn et al., who demonstrated that Lentulo spirals, as well as ultrasonic and sonic activation methods, achieved more effective sealer coating compared with manual K-file use and paper points (9).

### Apical extrusion of sealer, sealer volume, and duration of placement

An important procedural variable not evaluated by the included studies is apical extrusion of sealer during placement. While apical extrusion is clinically relevant because of its potential association with post-operative pain, delayed healing, and, for certain sealers, adverse tissue reactions, none of the included *in vitro* studies quantified or reported extruded sealer volume. The primary focus of the present review was on intracanal sealer layer characteristics, including sealer penetration into dentinal tubules, canal wall coverage, interfacial adaptation, void formation, and push-out bond strength, all of which directly reflect the quality of the sealer layer along the root canal walls. Consequently, extra-radicular outcomes such as apical sealer extrusion were beyond the scope of the available evidence synthesized in this review. Future studies should incorporate standardized assessment of apical extrusion alongside intracanal sealer layer characteristics to provide a more comprehensive evaluation of the clinical performance and safety of different sealer placement techniques.

In addition, the amount of sealer dispensed and the duration for which activation was applied were inconsistently reported and, where reported, varied considerably across the included studies. Both variables are known to influence sealer penetration depth and canal wall coverage, as larger sealer volumes and longer activation times may allow greater time for acoustic streaming to distribute sealer within the canal system before setting. The lack of standardisation of sealer volume and activation duration across studies represents a further source of methodological heterogeneity that may have contributed to the variability in pooled effect estimates and limits direct comparison of technique-related effects independent of these procedural parameters.

### Meta analysis

A formal meta-analysis was performed for sealer penetration depth and percentage of canal wall coverage with sealer; however, substantial statistical heterogeneity was observed across all pooled analyses (*I*^2^ ranging from 93.9% to 100.0%). The very high *I*^2^ values warrant careful interpretation, as *I*^2^ represents the proportion of total variability not attributable to sampling error and is therefore strongly influenced by the precision of the contributing studies. In standardised *in vitro* experiments, measurement conditions are tightly controlled and reported standard deviations are correspondingly small, resulting in very low sampling variance and consequently inflating *I*^2^ even when the absolute differences between studies are only moderate. In the present review, the typical sampling variance for penetration depth was 3.56 µm^2^, whereas the total heterogeneity (*τ*) was 109.05 µm, indicating that the absolute measure of dispersion (*τ*) provides a more informative assessment of between-study variability in this context than *I*^2^ alone.

The observed heterogeneity likely reflects true between-study differences in several factors rather than methodological noise alone, including sealer type (epoxy resin-based vs. calcium silicate-based formulations, which differ in rheological and physicochemical properties), ultrasonic activation protocols (tip design, frequency and power/amplitude settings, and duration of activation), the specific comparator technique evaluated (Lentulo spiral, hand file, or sonic activation), the canal level assessed (coronal, middle, or apical third), and the outcome assessment methodology used (e.g., CLSM vs. micro-CT). Furthermore, decomposition of the multilevel variance indicated that most heterogeneity arose from within-study rather than between-study differences (80.4% and 65.5% for penetration depth and canal wall coverage, respectively), reflecting the fact that individual experiments deliberately contributed multiple comparisons across different comparator techniques, canal levels, and sealer types. Moderator analysis further demonstrated that comparator technique alone explained approximately one-third to one-half of the total variance, indicating that a substantial proportion of the observed heterogeneity was systematic and clinically interpretable rather than random. Given this extreme heterogeneity, the pooled estimates likely reflect a distribution of substantially different underlying treatment effects rather than a single common effect and should therefore be interpreted as approximate, exploratory summaries rather than precise, generalisable estimates. This interpretation is reinforced by the 95% prediction intervals estimated for both outcomes. For the percentage of canal-wall coverage, the prediction interval (−7.45% to 43.04%) crossed the null, whereas the interval for penetration depth remained positive (28.54 to 480.77 µm) but was extremely wide. Together, these intervals describe the range within which the true effect of a comparable future study would be expected to lie and confirm that the pooled estimates summarise a genuinely dispersed, albeit partly explicable, distribution of effects rather than a single common treatment effect. Consequently, pooled WMD estimates should be interpreted with caution, as they represent a summary across heterogeneous conditions rather than a single, well-defined comparison. Although considerable heterogeneity remained, a substantial component could be explained by identifiable study characteristics, particularly the comparator technique, supporting the interpretation that the observed variability reflects systematic differences in experimental design rather than methodological inconsistency alone. The high heterogeneity may nevertheless influence the generalisability of the findings beyond the specific sealer and activation combinations evaluated in the included studies. Additionally, *in vitro* conditions cannot fully replicate the complex clinical environment, including moisture control, anatomical variability, and operator-related factors. Although improved sealer penetration and canal wall coverage may theoretically enhance the sealing ability of the obturation, direct evidence linking these surrogate outcomes to long-term clinical success remains limited; the translational relevance of these laboratory-based surrogate measures to clinical treatment outcomes has not yet been established and warrants dedicated clinical investigation. Within these constraints, the available evidence suggests that ultrasonic activation of root canal sealers enhances sealer layer quality across multiple outcome domains.

## Limitations

Despite the consistent trends favouring ultrasonic sealer placement, the findings of this review should be interpreted in light of several limitations. The number of included studies was limited, and considerable variation existed in tooth selection and root canal anatomy (including single-rooted teeth, canines, premolars, and individual molar roots), which may independently influence sealer distribution irrespective of the placement technique used. The comparator interventions themselves were heterogeneous, encompassing hand files, Lentulo spirals, and sonic devices with differing activation mechanisms, and irrigation protocols also varied across studies, which may have influenced smear layer removal and, consequently, sealer penetration. Outcome assessment methods also differed across studies (CLSM, micro-CT, stereomicroscopy, and mechanical push-out testing); these techniques vary in sensitivity and resolution, which limits direct comparability of findings across studies. All included studies were exclusively *in vitro*; this restriction reflects the fact that the primary outcomes evaluated in this review, sealer penetration depth, canal wall coverage, interfacial adaptation, and void formation, require destructive histological or microscopic analysis of sectioned root canals that cannot currently be performed in living patients.

Additionally, two included studies were authored by members of the present review team (11, 19). Both underwent the same QUIN-based risk-of-bias assessment as all other studies and were rated as low risk of bias. To assess the potential influence of author overlap, a sensitivity analysis excluding the self-authored study contributing to the meta-analysis (19) was performed. The pooled estimates remained consistent in direction, magnitude, and statistical significance, indicating that the findings were robust to its exclusion.

Certainty of evidence was not formally rated because GRADE has not been validated for *in vitro* research; instead, a structured appraisal adapted from the GRADE domains (Table 1) indicated very low overall confidence, primarily due to indirectness and inconsistency. Although the pooled estimates were robust to modelling of within-study dependency, the wider cluster-robust confidence intervals and the limited number of studies contributing to the between-study variance for penetration depth warrant cautious interpretation of the precision of the pooled estimates.

## Conclusion

Within the limitations of this systematic review, ultrasonic sealer placement techniques were associated with more favourable sealer layer quality compared with non-ultrasonic methods under standardised laboratory conditions. Ultrasonic activation was associated with deeper intratubular sealer penetration, more uniform canal wall coverage, improved interfacial adaptation, fewer voids, and greater push-out bond strength; however, the extremely high statistical heterogeneity observed across the pooled analyses (*I*^2^ = 93.9%–100.0%) indicates that these estimates summarise a range of substantially different underlying effects rather than a single precise effect, and the findings should therefore be regarded as exploratory rather than conclusive. As all included evidence was derived exclusively from *in vitro* studies conducted under standardised laboratory conditions, these results should not be interpreted as evidence of the clinical superiority of ultrasonic techniques. Well-designed clinical studies are required to determine the translational relevance of these laboratory findings and to establish whether the observed improvements in surrogate outcomes such as sealer penetration and canal wall coverage translate into improved long-term clinical success.

## Data Availability

The original contributions presented in the study are included in the article/Supplementary Material, further inquiries can be directed to the corresponding author/s.

## Supplementary material

Supplementary File S1 Search strategy: Detailed search strategy used across all electronic databases.

Supplementary File S2 Risk of bias: QUIN-based risk-of-bias assessment of the included studies.

Supplementary File S3 Data extraction chart: Summary of the methodological characteristics and key findings of the included in vitro studies, including sample details, sealer placement techniques, sealer type, assessment methods, evaluated outcomes, and principal results.

Supplementary File S4 Data for meta-analysis: Numerical dataset used for quantitative synthesis and meta-analysis.
