# Supplementary material for: Comparative effects of ultrasonic and conventional sealer placement techniques on endodontic sealer layer quality: a systematic review and meta-analysis of in vitro studies
Source: Front Dent Med. 2026 Aug 25;7:1909391. doi: 10.3389/fdmed.2026.1909391 (PMC13549913; doi:10.3389/fdmed.2026.1909391)
Supplement: Supplementary file 1 [file Table1.docx]

Search strategy

| **Search engine** | Search string | filters | Number of articles |
| --- | --- | --- | --- |
| Pubmed FIRST SEARCH | (("Root Canal Filling Materials"[MeSH] OR sealer* OR "root canal sealer*" OR "endodontic sealer*")  AND  ("root canal"[tiab] OR endodont* OR dentinal)  AND  (place* OR applic* OR distribut* OR delivery OR penetration OR void*)  AND  (lentulo OR ultrasonic OR sonic OR file OR instrument* OR "Max-i-Probe" OR "paper point")) | 2000-2025 Abstract, Full text, Books and Documents, Case Reports, Classical Article, Clinical Conference, Clinical Study, Clinical Trial, Clinical Trial Protocol, Clinical Trial, Phase I, Clinical Trial, Phase II, Clinical Trial, Phase III, Clinical Trial, Phase IV, Clinical Trial, Veterinary, Comparative Study, Evaluation Study, Observational Study, Randomized Controlled Trial, Technical Report, Validation Study, English, Exclude preprints. | 322 |
| Pubmed BROAD, HIGH-SENSITIVITY SEARCH | (  "Root Canal Filling Materials"[MeSH]  OR "Root Canal Obturation"[MeSH]  OR "endodontic sealer*"[tiab]  OR "root canal sealer*"[tiab]  OR sealer*[tiab]  )  AND  (  "root canal"[tiab]  OR endodont*[tiab]  )  AND  (  placement[tiab]  OR application[tiab]  OR delivery[tiab]  OR distribution[tiab]  ) | “ | 215 |
| Pubmed INSTRUMENT-FOCUSED SEARCH | (  "root canal sealer*"[tiab]  OR "endodontic sealer*"[tiab]  )  AND  (  lentulo[tiab]  OR "lentulo spiral"[tiab]  OR spiral[tiab]  OR file[tiab]  OR "K file"[tiab]  OR instrument*[tiab]  OR "bidirectional spiral"[tiab]  OR "Max-i-Probe"[tiab]  ) | “ | 91 |
| Pubmed ULTRASONIC / SONIC / ACTIVATION-BASED SEARCH | (  "root canal sealer*"[tiab]  OR "endodontic sealer*"[tiab]  )  AND  (  ultrasonic[tiab]  OR sonic[tiab]  OR activation[tiab]  OR vibratory[tiab]  OR oscillation[tiab]  )  AND  (  placement[tiab]  OR agitation[tiab]  OR penetration[tiab]  ) | “ | 4 |
| Pubmed OUTCOME-BASED SEARCH (VOID, PENETRATION, ADAPTATION) | (  "root canal sealer*"[tiab]  OR "endodontic sealer*"[tiab]  )  AND  (  void*[tiab]  OR penetration[tiab]  OR adaptation[tiab]  OR "sealer distribution"[tiab]  OR "dentinal tubule*"[tiab]  )  AND  (  placement[tiab]  OR technique*[tiab]  ) |  | 42 |
| Pubmed METHODOLOGY-FOCUSED (IMAGING-BASED STUDIES) | (  "root canal sealer*"[tiab]  )  AND  (  "micro computed tomography"[tiab]  OR micro-CT[tiab]  OR "confocal laser scanning microscopy"[tiab]  OR CLSM[tiab]  OR SEM[tiab]  )  AND  (  placement[tiab]  OR penetration[tiab]  OR void*[tiab]  ) |  | 19 |
| Pubmed COMPARATIVE STUDIES FILTER | (  "root canal sealer*"[tiab]  )  AND  (  placement[tiab]  OR technique*[tiab]  )  AND  (  compare*[tiab]  OR evaluat*[tiab]  OR effect*[tiab]  ) |  | 77 |
| Pubmed COMPREHENSIVE MeSH + FREE TEXT | **(**  **"Root Canal Filling Materials"[MeSH]**  **OR "Root Canal Obturation/methods"[MeSH]**  **OR "endodontic sealer*"[tiab]**  **OR "root canal sealer*"[tiab]**  **)**  **AND**  **(**  **lentulo[tiab]**  **OR ultrasonic[tiab]**  **OR sonic[tiab]**  **OR file[tiab]**  **OR instrument*[tiab]**  **OR spiral[tiab]**  **)**  **AND**  **(**  **penetration[tiab]**  **OR void*[tiab]**  **OR adaptation[tiab]**  **OR distribution[tiab]**  **)** |  | 146 |
| Scopus  FIRST SEARCH | **TITLE-ABS-KEY(**  **(sealer* OR "root canal sealer*" OR "endodontic sealer*")**  **AND**  **("root canal" OR endodont*)**  **AND**  **(placement OR delivery OR distribution OR penetration OR void* OR adaptation)**  **AND**  **(lentulo OR ultrasonic OR sonic OR file OR instrument* OR "Max-i-Probe" OR "paper point")**  **)** |  | 566 |
| Scopus  BROAD, HIGH-SENSITIVITY SEARCH (PRIMARY) | **TITLE-ABS-KEY (**  **( "root canal sealer*" OR "endodontic sealer*" OR sealer* )**  **AND**  **( "root canal" OR endodont* )**  **AND**  **( placement OR application OR delivery OR distribution )**  **)** |  | 658 |
| Scopus INSTRUMENT-SPECIFIC SEARCH | **TITLE-ABS-KEY (**  **( "root canal sealer*" OR "endodontic sealer*" )**  **AND**  **( lentulo OR "lentulo spiral" OR spiral**  **OR file OR "K file"**  **OR instrument***  **OR "bidirectional spiral"**  **OR "Max-i-Probe"**  **)**  **)** |  | 638 |
| Scopus ULTRASONIC / SONIC / ACTIVATION-BASED SEARCH | **TITLE-ABS-KEY (**  **( "root canal sealer*" OR "endodontic sealer*" )**  **AND**  **( ultrasonic OR sonic OR activation OR vibratory OR oscillat* )**  **AND**  **( placement OR agitation OR penetration )**  **)** |  | 70 |
| Scopus PROXIMITY-BASED (HIGH PRECISION) | **TITLE-ABS-KEY (**  **( "sealer" W/3 "placement"**  **OR "sealer" W/3 "application"**  **OR "sealer" W/3 "delivery"**  **)**  **AND**  **( "root canal" OR endodont* )**  **)** |  | 4 |
| Scopus OUTCOME-FOCUSED (VOID / PENETRATION / ADAPTATION) | **TITLE-ABS-KEY (**  **( "root canal sealer*" OR "endodontic sealer*" )**  **AND**  **( void* OR penetration OR adaptation OR "sealer distribution" )**  **AND**  **( technique* OR placement OR instrument* )**  **)** |  | 310 |
| Scopus IMAGING-BASED STUDIES (LAB-QUALITY FILTER) | **TITLE-ABS-KEY (**  **( "root canal sealer*" )**  **AND**  **( "micro-computed tomography" OR micro-CT**  **OR "confocal laser scanning microscopy" OR CLSM**  **OR SEM**  **)**  **)** |  | 336 |
| Scopus COMPARATIVE STUDIES FILTER | **TITLE-ABS-KEY (**  **( "root canal sealer*" )**  **AND**  **( placement OR technique* )**  **AND**  **( compare* OR evaluat* OR effect* )**  **)** |  | 588 |
| Scopus  MASTER SCOPUS SEARCH | **TITLE-ABS-KEY (**  **( "root canal sealer*" OR "endodontic sealer*" OR sealer* )**  **AND**  **( "root canal" OR endodont* )**  **AND**  **( placement OR application OR delivery OR distribution OR agitation )**  **AND**  **( lentulo OR ultrasonic OR sonic OR file OR instrument* OR spiral )**  **AND**  **( void* OR penetration OR adaptation OR quality )**  **)** |  | 111 |
| WOS BROAD, HIGH SENSITIVITY | **(("root canal sealer*" OR "endodontic sealer*" OR sealer*) AND ("root canal" OR endodont*) AND (placement OR application OR delivery OR distribution))** |  | 524 |
| WOS INSTRUMENT SPECIFIC | **(("root canal sealer*" OR "endodontic sealer*") AND (lentulo OR "lentulo spiral" OR spiral OR file OR "K file" OR instrument*))** |  | 453 |
| WOS ULTRASONIC / SONIC / ACTIVATION | **(("root canal sealer*" OR "endodontic sealer*") AND (ultrasonic OR sonic OR activation OR vibratory OR oscillation) AND (placement OR agitation OR penetration))** |  | 80 |
| WOS PROXIMITY SEARCH | **((sealer NEAR/3 placement OR sealer NEAR/3 application OR sealer NEAR/3 delivery) AND ("root canal" OR endodont*))** |  | 79 |
| WOS OUTCOME BASED | **(("root canal sealer*" OR "endodontic sealer*") AND (void* OR penetration OR adaptation OR distribution) AND (placement OR technique* OR instrument*))** |  | 320 |
| WOS IMAGE BASED STUDIES | **(("root canal sealer*") AND ("micro computed tomography" OR micro-CT OR "confocal laser scanning microscopy" OR CLSM OR SEM))** |  | 292 |
| WOS COMPARATIVE STUDIES | **(("root canal sealer*") AND (placement OR technique*) AND (compare* OR evaluat* OR effect*))** |  | 396 |
| WOS MASTER QUERY | **(("root canal sealer*" OR "endodontic sealer*" OR sealer*) AND ("root canal" OR endodont*) AND (placement OR application OR delivery OR distribution OR agitation) AND (lentulo OR ultrasonic OR sonic OR file OR instrument* OR spiral))** |  | 184 |
| WOS FIRST SEARCH | **(**  **(sealer* OR "root canal sealer*" OR "endodontic sealer*")**  **AND**  **("root canal" OR endodont*)**  **AND**  **(placement OR delivery OR distribution OR penetration OR void* OR adaptation)**  **AND**  **(lentulo OR ultrasonic OR sonic OR file OR instrument* OR "Max-i-Probe" OR "paper point")**  **)** |  | 482 |
